# Oxygen radical antioxidant capacity (ORAC) and antibacterial properties of *Melicope glabra* bark extracts and isolated compounds

**DOI:** 10.1371/journal.pone.0251534

**Published:** 2021-05-10

**Authors:** Alexandra Quek, Hafizah Mohd Zaini, Nur Kartinee Kassim, Fadzil Sulaiman, Yaya Rukayadi, Amin Ismail, Zamirah Zainal Abidin, Khalijah Awang

**Affiliations:** 1 Department of Chemistry, Faculty of Science, Universiti Putra Malaysia, Serdang, Selangor, Malaysia; 2 Integrated Chemical BioPhysic Research, Faculty of Science, Universiti Putra Malaysia, Serdang, Selangor, Malaysia; 3 Laboratory of Natural Products, Institute of Bioscience, Universiti Putra Malaysia, Serdang, Selangor, Malaysia; 4 Department of Dietetics, Faculty of Medicine and Health Science, Universiti Putra Malaysia, Serdang, Selangor, Malaysia; 5 Department of Oral Clinical Biology, Faculty of Dentistry, Universiti Kebangsaan Malaysia Kuala Lumpur, Kuala Lumpur, Malaysia; 6 Department of Chemistry, Faculty of Science, Universiti Malaya, Kuala Lumpur, Malaysia; Beijing Foreign Studies University, CHINA

## Abstract

*Melicope glabra* (Blume) T. G. Hartley from the Rutaceae family is one of the richest sources of plant secondary metabolites, including coumarins and flavanoids. This study investigates the free radical scavenging and antibacterial activities of *M*. *glabra* and its isolated compounds. *M*. *glabra* ethyl acetate and methanol extracts were prepared using the cold maceration technique. The isolation of compounds was performed with column chromatography. The free radical scavenging activity of the extracts and isolated compounds were evaluated based on their oxygen radical absorbance capacity **(**ORAC) activities. The extracts and compounds were also subjected to antibacterial evaluation using bio-autographic and minimal inhibitory concentration (MIC) techniques against two oral pathogens, *Enterococcus faecalis* and *Streptococcus mutans*. Isolation of phytoconstituents from ethyl acetate extract successfully yielded quercetin 3, 5, 3’-trimethyl ether (**1**) and kumatakenin (**2**), while the isolation of the methanol extract resulted in scoparone (**3**), 6, 7, 8-trimethoxycoumarin (**4**), marmesin (**5**), glabranin (**6**), umbelliferone (**7**), scopoletin (**8**), and sesamin (**9**). The study is the first to isolate compound (**1**) from Rutaceae plants, and also the first to report the isolation of compounds (**2–5**) from *M*. *glabra*. The ORAC evaluation showed that the methanol extract is stronger than the ethyl acetate extract, while umbelliferone (**7**) exhibited the highest ORAC value of 24 965 μmolTE/g followed by glabranin (**6**), sesamin (**9**) and scopoletin (**8**). Ethyl acetate extract showed stronger antibacterial activity towards *E*. *faecalis* and *S*. *mutans* than the methanol extract with MIC values of 4166.7 ± 1443.4 μg/ml and 8303.3 ± 360.8 μg/ml respectively. Ethyl acetate extract inhibited *E*. *faecalis* growth, as shown by the lowest optical density value of 0.046 at a concentration of 5.0 mg/mL with a percentage inhibition of 95%. Among the isolated compounds tested, umbelliferone (**7**) and sesamin (**9**) exhibited promising antibacterial activity against *S*. *mutans* with both exhibiting MIC values of 208.3 ± 90.6 μg/ml. Findings from this study suggests *M*. *glabra* as a natural source of potent antioxidant and antibacterial agents.

## 1. Introduction

The utilization of medicinal plants has surged in recent years owing to the presence of numerous biologically active secondary metabolites in these plants. Among these metabolites include polyphenols such as flavonoids, phenolic acids, lignans, and stilbenes. The bark of a medicinal plant has been considered as a significant source of bioactive phenolic compounds that possess excellent therapeutic properties [1]. For example, the bark of *Chloroxylon swietenia* was reported as a source of quercetin, ferulic acid, and gallic acid, essentially compounds that work as antioxidants and antiviral agents [2]. Meanwhile, gallotannin, ellagitannin and ampelopsin were found in the bark of *Anogeissus leiocarpa*, compounds of which exhibit antibacterial property [3]. Thus, the extraction of phenolic compounds from plants, especially from their bark, is of great interest to researchers. Maceration, microwave-assisted, and ultrasound-assisted extractions are among the different techniques used for the recovery of phenolic compounds from the bark of plants [1, 4].

Phenolics, carotenoids and anthocyanidins are plant-derived antioxidants that are effective in controlling excessive free radicals activity. Primary free radicals such as superoxide and hydroxyl radicals are generally associated with cell damage and apoptosis. In most cases, the abnormally high generation of free radicals causes an imbalance between free radicals and antioxidants in the body, and can lead to the development of various diseases [5]. The utilization of antioxidants is therefore considered in the management or treatment of oxidative stress-induced diseases including diabetes, stroke, cancer, myocardial infarction, and bacterial infections [6, 7]. For instance, carotenoids are suggested as chemopreventive agents while phenolic acids and flavones are proposed as antibacterial agents due to their potent antioxidant activity [7, 8].

Other than antioxidants, there have been numerous reports on the presence of antimicrobial agents in medicinal plants. An increasing number of medicinal plants have been reported for their potential application in the prevention or treatment of oral diseases including Brazilian folk medicine plant extracts which were tested against several oral pathogens including *Phorphyromonas gingivalis* and *Streptococcus mutans* [9]. From a previous study, two oxyprenylated acetophenones isolated from the Rutaceae family demonstrated potent antimicrobial activity in the prevention or treatment of common oral infections including dental caries [10].

*Melicope glabra* (Blume) T. G. Hartley has long been used in Indonesia as a traditional medicine in the treatment of fever, cough, and various infections [11]. It is an edible plant from the Rutaceae family and is commonly known as ‘setenggek burung’ among the locals in Malaysia. Previous reports on *Melicope glabra* revealed that the plant is enriched with phenolic compounds and exhibit promising antioxidant and anticancer activities [12, 13]. To date, there is very minimal research on the antibacterial properties of *Melicope glabra* against pathogenic oral bacteria, as well as a lack of data on the isolated bioactive compounds, although the *Melicope* species has been reported to exhibit a wide range of antimicrobial activities [9], an example being *Melicope ptelefolia* which was found to possess potent antibacterial and antifungal properties [14, 15]. In the realm of oral pathogens, *Streptococcus mutans* is regarded as the initiator of dental caries and is highly resistant towards many antibiotics [16], while *E*. *faecalis* is associated with the recurrent failure of endodontic treatment, forming colonies and biofilms even in medicated root canals of affected patients [17]. In this respect, this study aims to evaluate the potential of *M*. *glabra* extracts and its isolated compounds as a free radical scavenger and antibacterial agent against both *Enterococcus faecalis* and *Streptococcus mutans*. This study also highlights the isolation and characterization of the phytoconstituents obtained from *M*. *glabra* which may be responsible for the demonstrated biological activities.

## 2. Materials and methods

### 2.1. General procedures

1D-NMR; ^1^H and ^13^C, 2D-NMR; COSY, DEPT, HMQC, HMBC spectra were obtained by JEOL JNM CRX 400MHz or JEOL JNM ECX 500MHz spectrometers in chloroform-d (CDCl_3_) and acetone-d6 (CD_3_COCD_3_) using tetramethylsilane (TMS) as the internal standard. The Infrared Spectroscopy (IR) spectra were recorded using KBR discs on a Perkin Elmer FTIR spectrometer model 1275X. Gas Chromatography-mass spectroscopy (GC-MS) was carried out on a QP5050A SHIMADZU equipped with BPX5 for non-polar (5% phenyl methylsiloxane) capillary column with a dimension of 30.0 m X 250 μm X 0.25 μm. GC oven temperature was programmed from 50 to 265⁰C at a rate of 5⁰C min^-1^ with an initial hold of one minute and hold of ten minutes, whereby the MS was operated with the ionization induced by electron impact at 70eV. The compounds were subjected to direct injection techniques to separate all the components in the sample for a representative spectral output. Ultraviolet (UV) spectra were obtained on a Shimadzu UV-2100 spectrophotometer. The melting point of the compounds were determined using a Leica Galen III microscope equipped with a Testo 720 temperature recorder. The bioassay for antimicrobial work was conducted under a laminar flow ESCO EQU-04. The apparatus, broth, agar and utensils used for the antimicrobial assay were autoclaved in a HIRAYAMA Autoclave HVE50. The incubator used for the bioassay works was a IB-05G Jeio Tech under 36/37⁰C. A glycerol stock of bacteria was frozen and stored in a Haier UT Freezer at -80⁰C. The tested sample tested was read by a Thermofisher Varioskan Flash at 625 nm wavelength. A FLUOstar OPTIMA microplate fluorescence reader (BMG LABTECH, Offenburg, Germany) was used for the determination of oxygen radical absorbance capacity (ORAC) value.

### 2.2. Chemical and reagents

Column chromatography (CC) was carried out using silica gels Merck Kieselgel PF254 Art. No. 1.007749.1000, 60 Art. No. 9385.1000 and No. 1.07734.1000. Deuterated solvent (chloroform, methanol, and acetone) were purchased from Sigma Aldrich (St. Louis, USA). Thin layer chromatography (TLC) was performed using commercially available Merck DC-Plasticfolien TLC plastic sheet pre-coated with Kieselgel 60 PF₂₅₄ (0.2 mm thickness). Brain heart infusion (BHI) broth, and dimethylsulfoxide (DMSO) were obtained from Sigma Aldrich. The bacteria strains used were ATCC 29212 *Enterococcus faecalis* (USA) and KCCM3309 *Streptococcus mutans* (Daejeon, South Korea). α-Tocopherol, ascorbic acid and butylated hydroxytoluene (BHT) acted as positive controls. Reagents used for the ORAC assay, 2,2’-Azobis(2-amidino-propane) dihydrochloride (AAPH), fluorescein, and 6-hydroxy-2,5,7,8-tetra-methylchroman-2carboxylic acid (Trolox) were prepared in 75 mM phosphate buffer (pH 7.4).

### 2.3. Plant materials

The stem bark of *Melicope glabra* (1.0 kg) was collected from an 18 m tall *Melicope glabra* tree in Kedah, Malaysia on 6^th^ of March, 1996. The plant was identified by Mr. Teo L. E. and Tarelli O., and a voucher specimen numbered 4563 was deposited in the Department of Biology, University of Malaya, Malaysia.

### 2.4. Extraction and isolation

The extraction of *M*. *glabra* was performed via the cold maceration technique as described in a previous study [13]. The dried stem bark (1.0 kg) was first ground to course powder and subjected to extraction cold maceration using two solvents of increasing polarity (ethyl acetate and methanol). The extract was filtered after 72 hours and evaporated to dryness using a rotary evaporator under reduced pressure. The soaking process was repeated for another two times to maximize the yield. Ethyl acetate and methanol crude extracts weighing 36 g and 39 g respectively were obtained.

The ethyl acetate extract (17 g) was subjected to a column chromatography (CC) fractionation. The column (8.5 cm × 20 cm) was eluted with different solvent system in increasing polarity order starting from hexane, chloroform, ethyl acetate, and methanol to produce sixty-eight fractions of 200 ml each. The washing and rinsing of fractions 24–27 with a hexane solvent yielded compound **1** (12 mg) in the form of yellow needle crystals. The separation of fractions 28–32 by CC (1.5 cm × 25 cm) with a mixture of mobile phases (hexane:chloroform:ethyl acetate:methanol) gave seventy-one subfractions. Subfraction 24 underwent further purification using chromatotron with a gradient solvent system of hexane:chloroform:ethyl acetate which yielded kumatakenin (**2**) (8 mg) as yellow needle crystals.

Next, 15 g of the methanol extract was subjected to a dry vacuum CC separation using a combination of solvents, namely hexane, ethyl acetate and methanol. Thirty eight fractions of 200 mL each were obtained, fractions 12–15 and 16–17 were combined and the re-chromatography of fractions 12–15 which were eluted using a hexane:chloroform gradient mixture resulted in scoparone (**3,** 2 mg) and 6, 7, 8-trimethoxycoumarin (**4,** 2 mg). Meanwhile, the re-chromatography of fractions 16–17 eluted with the same mixture of hexane:chloroform gave 45 sub-fractions of 50 mL each. Further separation on sub-fractions 32–36 led to the isolation of marmesin (**5,** 12 mg). The isolation and characterization of glabranin (**6**, 6 mg), umbelliferone (**7**, 100 mg), scopoletin (**8**, 27 mg) and sesamin (**9**, 210 mg) was previously described by Kassim et al. [13].

### 2.5. Characterization of isolated compounds

Characterization of the isolated compounds was carried out using nuclear magnetic resonance (NMR), mass spectroscopy (MS), ultraviolet spectroscopy (UV-Vis), and infrared spectroscopy (IR). One-dimensional (1D) (^1^H, ^13^C) and two-dimensional (2D) (COSY, HMQC, HMBC, DEPT) NMR spectra of the pure compounds were processed with the MestReNova software ver. 14.1.2 (Mestrelab Research S.L., Santiago de Compostela, Spain). The spectroscopic data of the known compounds was compared with that of existing literature.

### 2.6. Determination of Oxygen radical absorbance capacity (ORAC)

ORAC evaluation of extracts and isolated compounds follows a method described in a previous study [13]. 2,2’-azobis (2-amidino-propane) dihydrochloride (AAPH), fluorescein, and 6-hydroxy-2,5,7,8-tetra-methylchroman-2-carboxylic acid (trolox) were prepared in 75 mM phosphate buffer with a pH of 7.4. An excitation wavelength of 485 nm and an emission wavelength of 520 nm were applied in the assay, whereby the ORAC value was referred to as the net protective area under the quenching curve of fluorescein in the presence of an antioxidant. The net area under the curve (AUC) of the standard and samples were calculated. The antioxidant capacity (ORAC) related to trolox was calculated as follows:

$$ORAC\;value=[(AUC_{sample}‐AUC_{blank})/(AUC_{Trolox}‐AUC_{blank})][trolox]\;dilution\;factor$$

The results were evaluated with the aid of the MARS software and expressed as μmol TE per gram of sample.

### 2.7. Antibacterial activity

#### 2.7.1. Preparation of bacteria

The bacteria of choice were *Enteroccocus faecalis* (ATTC 29212) and *Streptococcus mutans* (KKCM3309). Both bacteria were subcultured and a colony was transferred into a universal bottle containing 10 mL of broth. The inoculum was then incubated at 37⁰C for 24 hours. The turbidity of the inoculum was adjusted to 0.5 McFarland (~0.8–1.0 × 10^8^ CFU/mL) and diluted to 10^6^ CFU/mL prior to subsequent experiments.

#### 2.7.2. TLC-bioautography assay: Spraying and dipping

Two techniques of direct TLC-bioautography (spraying and dipping methods) were used for the screening of antibacterial compounds in the extracts [18]. The TLC-bioautography was performed according to a method described by Mosoko [5] with slight modifications. For the spraying method, a fine glass capillary tube was used to spot the sample onto the TLC sheets and then developed in the TLC chamber using a solvent system of *n*-hexane:ethyl acetate (3:2) for the ethyl acetate extract and chloroform:methanol (5:few drops) for the methanol extract, both of which were conducted at room temperature. The TLC spots were observed under UV light (254 or 360 nm wavelength) and placed on a dry Petri dish before the bacteria culture of 10^6^ CFU/mL was sprayed onto the sheet and incubated at 37⁰C for 24 hours. The MTT solution was then sprayed onto the bioautogram and observed after 2 hours. Meanwhile the dipping method was carried out by dipping the developed TLC sheets in 10^6^ CFU/mL of bacteria culture. The absence of purple discoloration from the MTT dye indicates the inhibition of bacterial growth by the separated compounds.

#### 2.7.3. Minimal inhibitory concentration (MIC) assay

The minimal inhibitory concentration (MIC) assay was carried out following a method described by Park et al. [19] with some modifications. Firstly, 10 mg of each crude extract and isolated compound was dissolved in 1 mL of 0.1% DMSO, then 50 μL of the sample was pipetted into the sample wells of a sterile 96-well microplate and serially diluted (10000–39.1 μg/mL) before adding 50 μL of 10^6^ CFU/mL bacteria culture into each of the well. Amoxicillin at a concentration of 100 μg/mL was used as a positive control. A broth mixture without any inoculum but with plant extracts and another broth mixture containing the inoculum mixed in 0.1% DMSO were also included as negative controls. The experiment was done in triplicates. Subsequently, the plates were incubated at 37 ⁰C for 24 hours and observed using a plate reader at 625 nm wavelength.

### 2.8. Statistical analysis

All experiments were carried out in triplicates. The results were expressed as mean ± standard deviation and differences between means were statistically analyzed using the t-test to compare two treatments, with p < 0.05 considered significant. The statistical analysis was performed with GraphPad Prism (San Diego, CA).

## 3. Results and discussion

### 3.1. Isolation of compounds from *M*. *glabra* extracts

**Fig 1:**
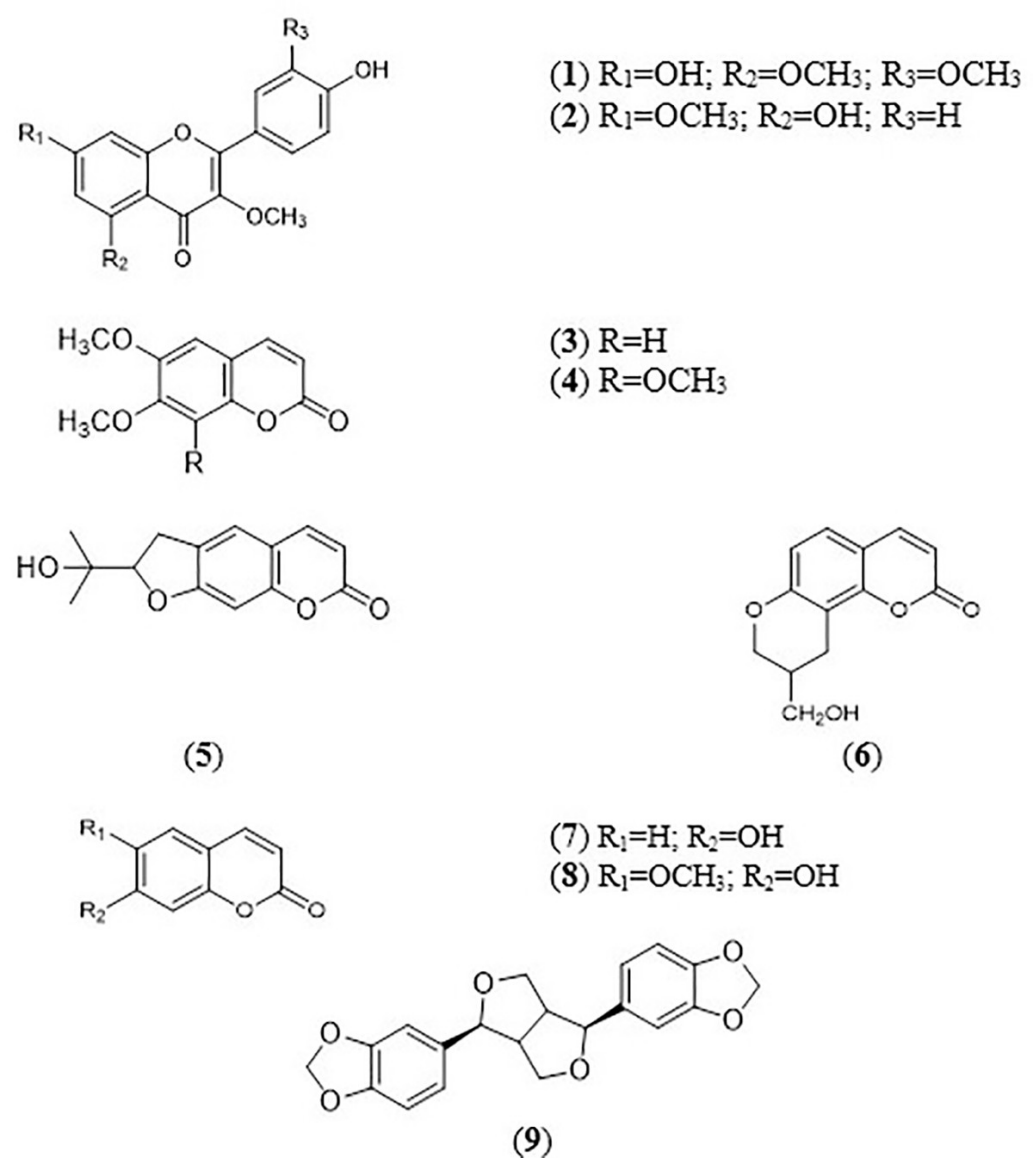
Structures of compounds 1–9 isolated from *M*. *glabra* ethyl acetate and methanol extracts.

The column chromatrography of the ethyl acetate extract of *M*. *glabra* resulted in the isolation of quercetin 3, 5, 3’-trimethyl ether (**1**) [20] and kumatakenin (**2**) [21]. The isolation of the methanol extract of *M*. *glabra* afforded scoparone (**3**) [22], 6, 7, 8-trimethoxycoumarin (**4**) [23], marmesin (**5**) [24], glabranin (**6**) [13], umbelliferone (**7**) [25], scopoletin (**8**) [26] and sesamin (**9**) [27]. The structures of the compounds were established by detailed spectroscopy analyses including ultraviolet (UV), mass spectroscopy (MS), infrared (IR) and nuclear magnetic resonance (1D and 2D-NMR) spectral data, and the data compared with existing literature. Among the compounds obtained, the isolation of compounds (**6**–**9**) from *M*. *glabra* was previously reported [12]. To the best of our knowledge, this study is the first to describe the extraction of compounds (**2–5**) from *M*. *glabra*, while this study is the first to isolate compound (**1**) from Rutaceae plants. The mass spectrum of compounds **1**–**9** can be found in S1–S9 Figs. Fig 1 shows the structures of the isolated compounds.

### 3.2. Determination of Oxygen radical absorbance capacity (ORAC)

**Fig 2:**
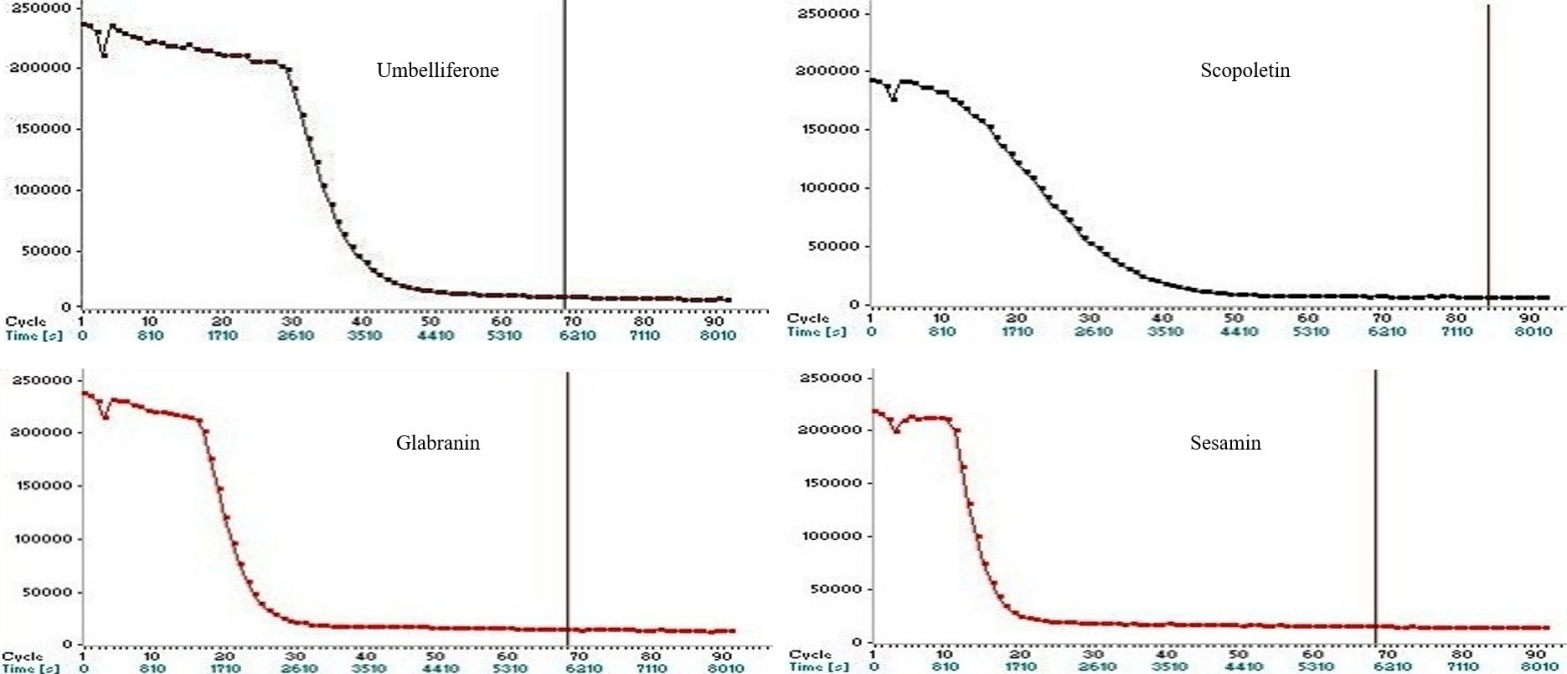
Fluorescence decay curves of fluorescein induced by AAPH in the presence of umbelliferone (7), glabranin (6), scopoletin (8) and sesamin (9).

The potential of a compound to scavenge peroxyl radicals initiated by spontaneous decomposition of 2,2’- azo-bis, 2- amidinopropane dihydrochloride (AAPH) was evaluated in terms of standard equivalents [28]. The spectrophotometric ORAC assay on the methanol and ethyl acetate extracts demonstrated good antioxidant activity with their respective values of 272760.4 ± 3294.3 and 190160.2 ± 1126.4 μmolTE/g [11] while glabranin (**6**), umbelliferone (**7**), scopoletin (**8**) and sesamin (**9**) resulted in ORAC values of 2883.0 ± 209.7, 24965.1 ± 1943.2, 2007.1 ± 225.8, and 2319.7 ± 132.8 μmolTE/g respectively (Table 1). Umbelliferone (**7**) exhibited the highest ORAC value among the tested compounds indicating its potential as a free-radical scavenger as illustrated in Fig 2 which showed the inhibition of fluoresence decay during the quenching of AAPH. The strength of the scavenging activity from the ORAC assay can be presented in the order of: methanol extract > ethyl acetate extract > umbelliferone (**7**) > glabranin (**6**) > sesamin (**9**) > scopoletin (**8**).

**Table 1:** Biological activities of *M*. *glabra* crude extracts and their isolated compounds.

Sample	ORAC μmolTE/g	Antimicrobial activity MIC (μg/mL)	
		*E*. *faecalis*	*S*. *mutans*
Ethyl acetate crude	190160.2 ± 1126.4 ^b^	4166.7 ± 1443.4 ^c^	833.3 ± 360.8 ^c^
Methanol crude	272760.4 ± 3294.3 ^a^	8 333.3 ± 2 886.8 ^d^	1 041.7 ± 360.8 ^d^
Glabranin (**6**)	2883.0 ± 209.7 ^d^	ND	ND
Umbelliferone (**7**)	24965.1 ± 1943.2 ^c^	ND	208.3 ± 90.6 ^b^
Scopoletin (**8**)	2007.1 ± 225.8 ^e^	ND	ND
Sesamin (**9**)	2319.7 ± 132.8 ^e^	1 041.7 ± 360.8 ^b^	208.3 ± 90.6 ^b^
Amoxicillin	-	100 ± 0 _a_	100 ± 0 ^a^

* ‘-’: Not tested. ND-Not determined even when tested at highest concentration (10,000 μg/mL). Data with a different superscript in the same column were considered significant (p < 0.05).

Paya et al. [29] reported that both umbelliferone (**7**) and scopoletin (**8**) exhibited scavenging capabilities towards hydroxyl radicals and superoxide anions in the hypoxanthine-xanthine oxidase system. Furthermore, umbelliferone (**7**) was also shown to be able to restrain prostaglandin biosynthesis while displayed a strong activity in 2,2-diphenyl-1-picrylhydrazyl (DPPH) and thiobarbituric acid (TBA) lipid peroxidation assays [30]. 5, 6, 7-Trimethoxycoumarin (**4**), on the other hand, exhibited good antiglycation activity in a previous study [31]. Unfortunately, further ORAC tests could not be carried out on the other compounds due to the inadequate quantity of samples available.

### 3.3. Antibacterial activity

#### 3.3.1. TLC-bioautography assay of crude extracts

**Fig 3:**
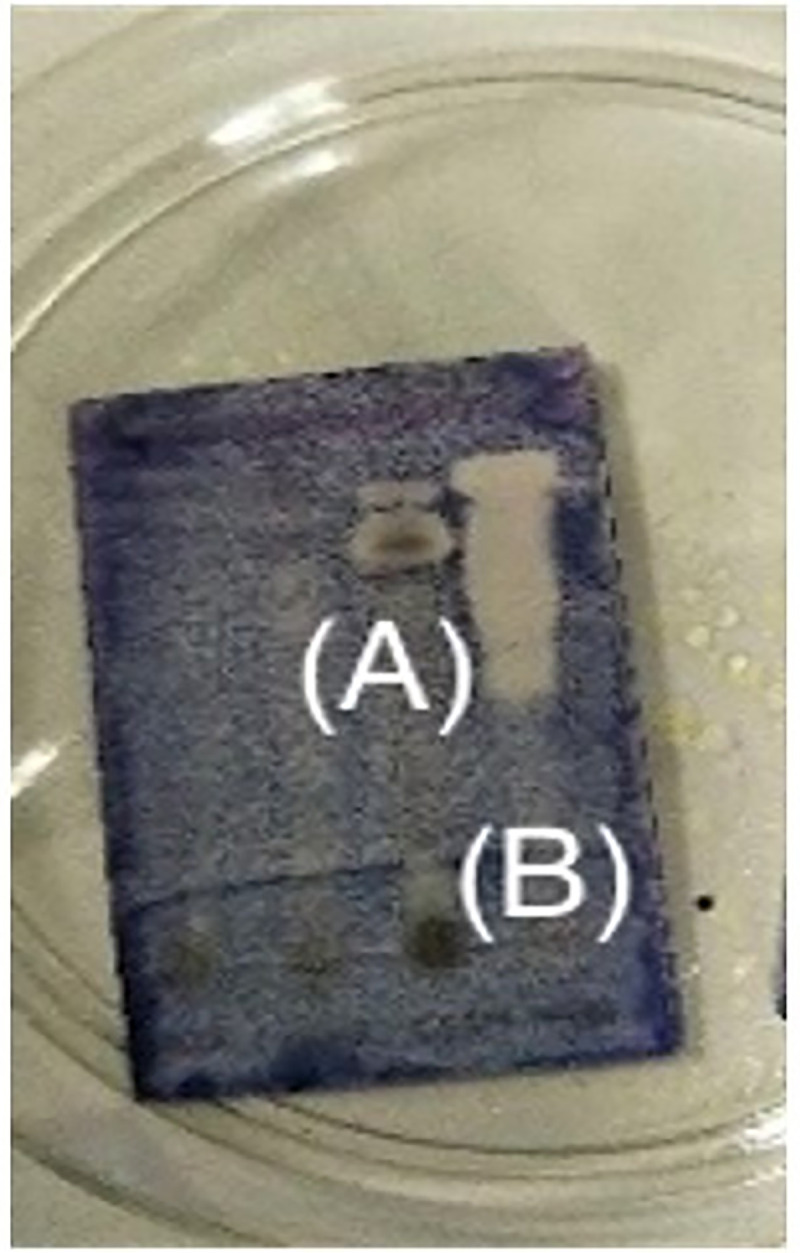
TLC-bioautography of the methanol extract (A) and ethyl acetate extract (B) after spraying with *E*. *faecalis* bacterial culture.

TLC-bioautography assay was carried out to qualitatively investigate the antibacterial activity of the *M*. *glabra* crude extracts. The appearance of a white region or clear zone on the MTT-stained TLC indicated the absence of bacterial growth. Due to the appearance of a clear zone on the stained TLC plate as shown in Fig 3, it is suggested that both the ethyl acetate and methanol crude extracts exhibit antibacterial activity against *E*. *faecalis*.

#### 3.3.2. Minimal inhibitory concentration (MIC) of crude extracts and isolated compounds

The minimal inhibitory concentration (MIC) assay is a widely used and accepted quantitative method in measuring the sensitivity of an organism towards its inhibitors based on the contact of the test organism to a serially diluted test substance [32]. MIC is defined as the lowest concentration of sample which gives rise to complete bacterial growth inhibition and the mean value from three independent replicates is usually taken [33].

In this study, the antibacterial efficacy of *M*. *glabra* extracts as well as their isolated compounds were further evaluated based on their MIC values tested against *S*. *mutans* and *E*. *faecalis*. Table 1 shows that the ethyl acetate extract exhibited stronger antimicrobial activity against *E*. *faecalis* and *S*. *mutans* with MIC values of 4166.7 ± 1443.47 μg/mL and 833.3 ± 360.8 μg/mL respectively compared to the methanol extract. The results are consistent with the TLC-bioautography screening in which the ethyl acetate extract exhibited a broader inhibition area than the methanol extract against *E*. *faecalis*. The MIC results also indicate that the ethyl acetate extract is more effective against *S*. *mutans* compared to *E*. *faecalis*. A study reported that plant extracts of lower polarity from the Labiatae, Liliaceae, and Zingiberaceae families exhibit good antibacterial activity towards *S*. *mutans* [34]. The primary acidogenic components of dental biofilm produced by *S*. *mutans* is able to metabolize exogenous dietary carbohydrates into lactic acid, leading to the demineralization of tooth enamel. The first clinical sign of periodontal disease is the colonization shift towards facultative and anaerobic bacterial species which will result in supragingival plaque aggregation and gingival infections as the dental biofilm matured. This includes *E*. *faecalis* which are associated with the persistent infection of the root canals, resulting in treatment failure [16]. Therefore, targeted approach towards the inhibition of *S*. *mutans* growth may halt the emergence and spread of *E*. *faecalis*.

The isolated compounds umbelliferone (**7**) and sesamin (**9**) show good inhibitory activity against *S*. *mutans* with MIC values of 208.3 ± 90.6 μg/mL which were better than the crude extracts. The positive control, amoxicillin exhibit MIC value of 100 ± 0 μg/ml against *S*. *mutans*. The strength of the antibacterial activity against *S*. *mutans* can be ranked as follows: umbelliferone (**7**) and sesamin (**9**) > ethyl acetate extract > methanol extract. Due to the limited amount of isolated compounds available for use, it was not possible to perform further MIC assays. Bonifait et al. observed that oxyprenylated secondary metabolites extracted from Rutaceae family showed good antimicrobial activity against oral pathogens including gram-positive bacteria (*Streptococcus mutans*, *Streptococcus sobrinus*), gram-negative bacteria (*Prevotella intermedia*, *Porphyromonas gingivalis*) and *Candida albicans* [10]. This suggests that natural products from plants can be used as a lead compound in the development of drugs for the prevention and treatment of dental caries and periodontal disease.

## 4. Conclusion

This study demonstrated that *M*. *glabra* extracts and isolated compounds possess promising antioxidant and antibacterial properties with umbelliferone (**7**) exhibiting the strongest antioxidant activity and both umbelliferone (**7**) and sesamin (**9**) demonstrating the highest antibacterial activity against *S*. *mutans*. This study can be expanded towards the development of a plant-based mouthwash formulation as a preventive aid in reducing oral plaque formation and gingival inflammation. Further *in vivo* studies on the antioxidant and antibacterial activities of *M*. *glabra* extracts and bioactive compounds are also recommended.

## Supporting information

S1 Fig EIMS of quercetin 3, 5, 3’-trimethyl ether (1). (DOCX)

S2 Fig EIMS of kumatakenin (2). (DOCX)

S3 Fig EIMS of scoparone (3). (DOCX)

S4 Fig EIMS of 6, 7, 8-trimethoxycoumarin (4). (DOCX)

S5 Fig EIMS of marmesin (5). (DOCX)

S6 Fig EIMS of glabranin (6). (DOCX)

S7 Fig EIMS of umbelliferone (7). (DOCX)

S8 Fig EIMS of scopoletin (8). (DOCX)

S9 Fig EIMS of sesamin (9). (DOCX)
