# Supplementary figures and images for: Oxygen radical antioxidant capacity (ORAC) and antibacterial properties of Melicope glabra bark extracts and isolated compounds
Source: PLoS One. 2021 May 10;16(5):e0251534. doi: 10.1371/journal.pone.0251534 (PMC8109830; doi:10.1371/journal.pone.0251534)

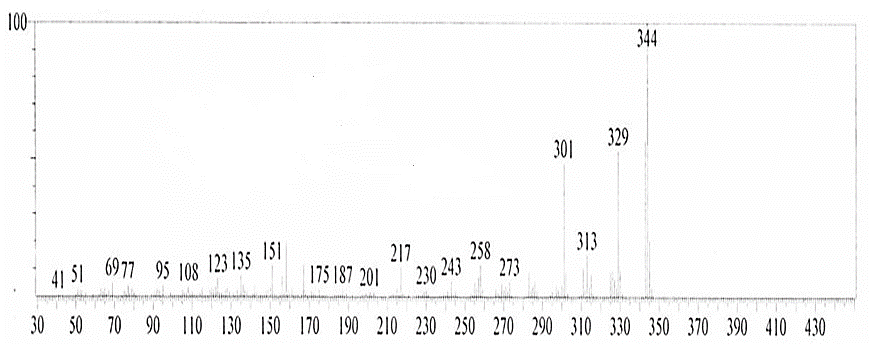


S1 Fig. EIMS of quercetin 3, 5, 3'-trimethyl ether (**1**).

Supplement: S1 Fig — (DOCX) [file pone.0251534.s001.docx]

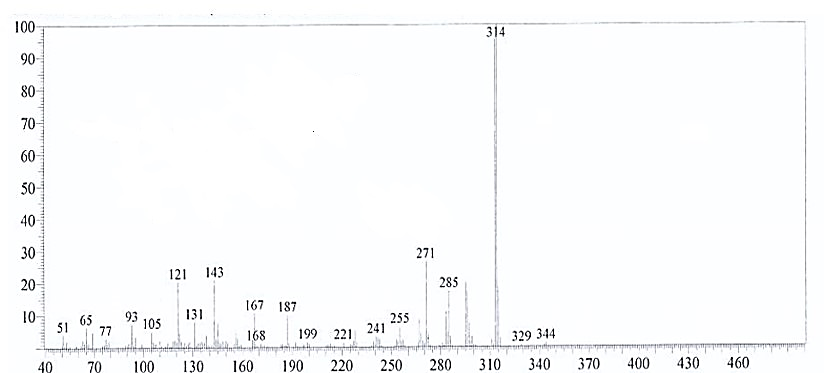


S2 Fig. EIMS of kumatakenin (**2**).

Supplement: S2 Fig — (DOCX) [file pone.0251534.s002.docx]

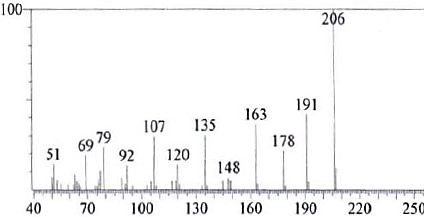


S3 Fig. EIMS of scoparone (**3**).

Supplement: S3 Fig — (DOCX) [file pone.0251534.s003.docx]

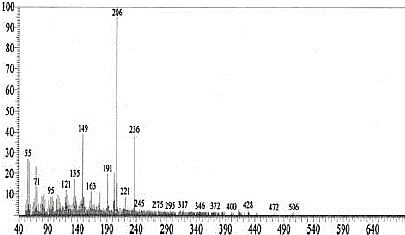


S4 Fig. EIMS of 6, 7, 8-trimethoxycoumarin (**4**).

Supplement: S4 Fig — (DOCX) [file pone.0251534.s004.docx]

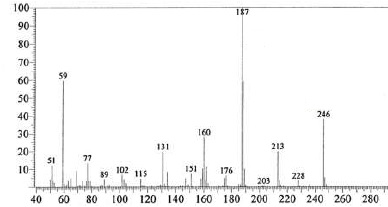


S5 Fig. EIMS of marmesin (**5**).

Supplement: S5 Fig — (DOCX) [file pone.0251534.s005.docx]

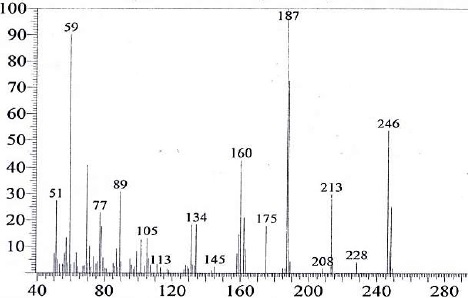


S6 Fig. EIMS of glabranin (**6**).

Supplement: S6 Fig — (DOCX) [file pone.0251534.s006.docx]

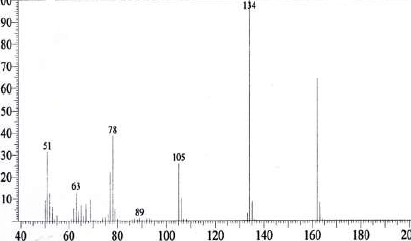


S7 Fig. EIMS of umbelliferone (**7**).

Supplement: S7 Fig — (DOCX) [file pone.0251534.s007.docx]

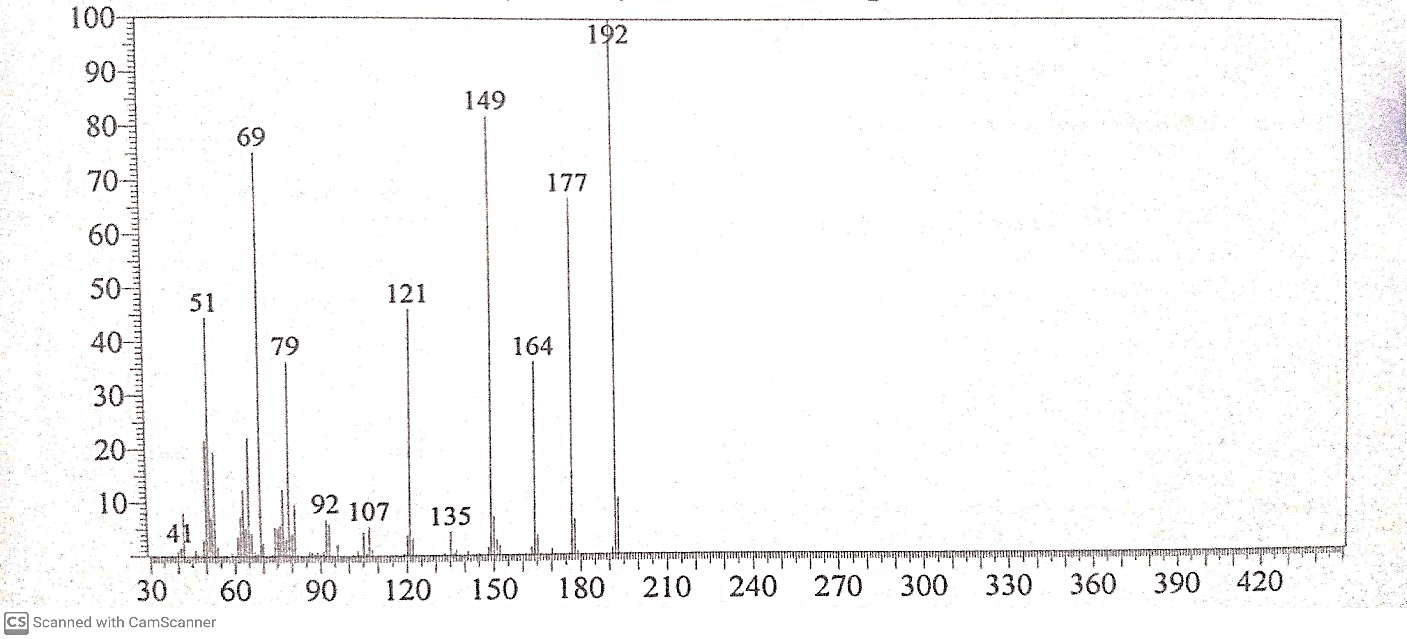


S8 Fig. EIMS of scopoletin (**8**).

Supplement: S8 Fig — (DOCX) [file pone.0251534.s008.docx]

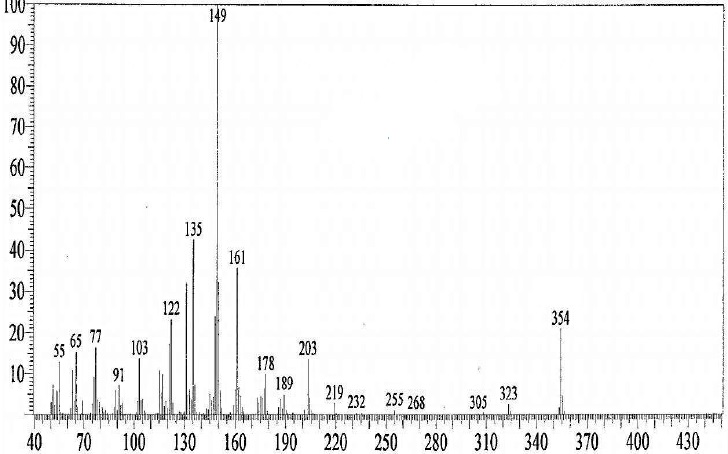


S9 Fig. EIMS of sesamin (**9**).

Supplement: S9 Fig — (DOCX) [file pone.0251534.s009.docx]
